# Volumetric Assessment of Blow-Out Fractures With Automated Segmentation Benefits Thinner Computed Tomography Slice Thickness: A Retrospective Case-Control Study

**DOI:** 10.1097/SCS.0000000000012681

**Published:** 2026-04-13

**Authors:** Eeva Kormi, Niilo Lusila, Pilvi Mäntynen, Ville Männistö, Juho Suojanen

**Affiliations:** * Department of Oral and Maxillofacial Surgery, Päijät-Häme Central Hospital, Päijät-Häme Joint Authority for Health and Wellbeing; † Faculty of Medicine and Clinicum, University of Helsinki; ‡ Department of Radiology, Päijät-Häme Central Hospital, Päijät-Häme Joint Authority for Health and Wellbeing, Lahti; § Department of Plastic Surgery, Cleft Palate and Craniofacial Centre, Helsinki University Hospital, Helsinki, Finland

**Keywords:** Automated segmentation, blow-out fracture, computed tomography, medical modelling, orbital volume

## Abstract

The volume of the orbital vault is an important factor in assessing asymmetry, blow-out fractures, and postoperative outcomes after reconstruction. However, manual segmentation is time-consuming and may depend on the radiologist's experience. The automated segmentation tool speeds up the process, but it may not yield results as similar to manual segmentation. The orbital volume of 34 patients suffering from orbital blow-out fracture requiring surgical intervention and pre- and postoperative computed tomography (CT) data were assessed with an automated segmentation tool (case) and manual segmentation (control). Pre- and postoperative CTs of fractured and intact orbits were assessed (n=136). Slice thickness as a factor affecting the repeatability results of automated segmentation was analysed with the Mann-Whitney *U* test. Agreement between methods and measurements was assessed using the interclass correlation coefficients (ICC), and the repeatability of each method was tested using intraclass correlation coefficients (ICC). Compared with thicker (2 mm+) slices, 1 mm or less slice thickness showed significantly better repeatability between pre- and postoperative imaging when automated segmentation was used (*P*=0.028), although overall repeatability was very good in both methods; ICC was 0.83 in manual segmentation and 0.92 in automated segmentation. Correlation between automated and manual segmentation was rather poor in both intact and fractured orbits in pre- and postoperative imaging (ICC: 0.57 and 0.50 in intact orbits; 0.41 and 0.67 in fractured and reconstructed orbits). Automated segmentation is a reliable and repeatable method in volumetric assessment, especially with a slice thickness of 1 mm or less. However, volumetric results do not correlate with manual segmentation.

The fractures of the bony orbital floor, also called blow-out fractures, are common in facial trauma.^1^ The complex anatomy and delicate structures of the orbit are impossible to assess fully with 2-dimensional (2D) imaging, and the gold standard for diagnostics is computed tomography (CT),^2^ but if osseous anatomy is the only interest, cone beam CT (CBCT) can be used as well.^3^ Whether to operate or not is a multifactorial decision and should not be done without clinical assessment^4,5^ but enlarged volume of the orbital vault is one important factor.^6,7^ Fracture of the zygomatic bone can also affect the volume of the bony orbit and may sometimes lead to the need for orbital floor reconstruction; however, the decision is even more complicated since the reposition of the zygomatic bone alone may be sufficient to correct the orbital floor displacement as well. For this reason, isolated orbital floor fractures must be evaluated as distinct entities. The normal orbital volumes are bilaterally rather symmetric,^8–11^ and the contralateral side can be used for reference. The 3-dimensional (3D) imaging is also used for virtual surgical planning (VSP) and reconstruction of blow-out fractures, as patient-specific implants (PSI) are becoming more common.^12–14^ For blow-out fracture reconstruction, the contralateral side can be mirrored.^15^ The 3D imaging can also be used for medical modelling, and a 3D printed model of the fractured orbit can be used as a template when moulding titanium mesh for reconstruction.^16^

For volumetric analysis of the orbit, a radiologist can use manual segmentation, in which the bony walls of the orbit are defined slice by slice to form a 3D image of the vault and assess its geometry and volume. This is rather time-consuming, and even this may contain inaccuracies, though measurements of bony structures are more reliable.^17^ Novel technologies are making it possible to semi-automate this process.^18,19^

The aim of this study is to assess the repeatability and reliability of the automated segmentation tool for measuring intact and fractured bony orbits, compared with manual segmentation by a radiologist, with emphasis on the effect of CT slice thickness on automated method repeatability.

## METHODS

This was a retrospective study of patients of Päijät-Häme Central Hospital, Lahti, Finland, from 2008 to 2020. The research adhered to the principles outlined in the Declaration of Helsinki. The study protocol was approved by Päijät-Häme Central Hospital (Permission D/18/07.01.04.05/2018 and D/2929/07.01.04.05/2020). All patients with facial fractures (ICD10 diagnosis code S02.XX) were drawn from the patient registry and evaluated for blow-out fractures. Inclusion criteria were patients suffering from unilateral blow-out fracture requiring surgical intervention and with CT or CBCT of orbits taken preoperatively and postoperatively. Patients with other simultaneous facial fractures were excluded.

**FIGURE 1:**
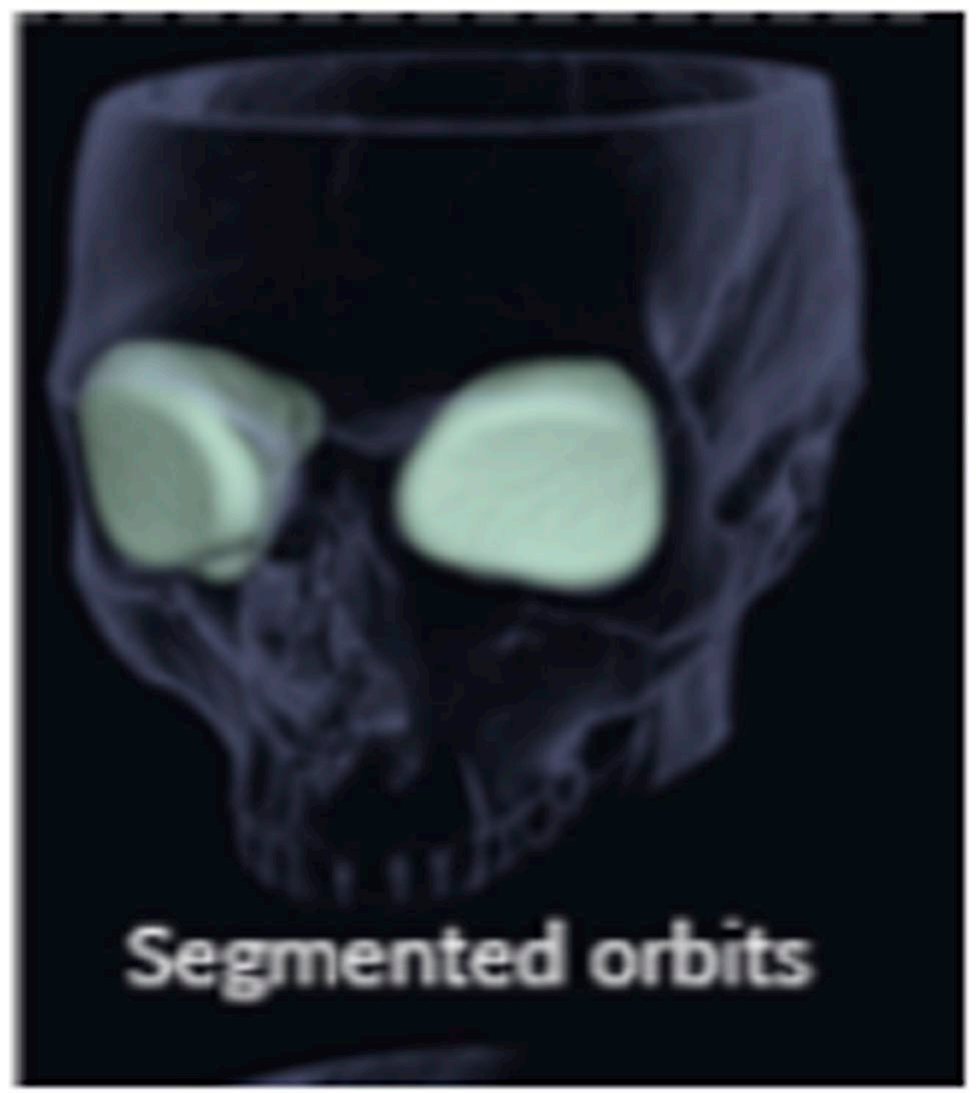
After importing the DICOM files, the automated segmentation tool quantifies the affected (right) and unaffected (left) orbits.

The preoperative and postoperative CTs were analysed with an automated segmentation tool and manually by the same radiologist (N.L.). For automated segmentation, the volume of the bony orbit was measured using (CMF Orbital Software; Disior Ltd., Helsinki, Finland) (Fig. 1) as described earlier.^9,13^

For software analysis, the DICOM data is imported into software and converted to a voxel map. The radiologist chooses the side to be examined and selects a seed point anywhere inside the orbital cavity. In this study, the seed point was selected at the apex of the orbit, at the junction of the optic nerve and bulbus, in all cases, although this selection should not affect the results. The program works fully automatically after this, and from the seed point, the triangle mesh iteratively expands until it self-limits on the bony walls of the orbit or anteriorly on the bony rims. At the fracture site, the shortest distance between the fragment rims and the remaining floor rims limits the expanding mesh. The automated analyses and the software algorithm's surface reliability were confirmed by the radiologist in all cases. For manual volume measurement, the radiologist segmented the orbit slice by slice, defining its bony walls, and used 3D segmentation and volume-measurement tools. Volumetric analyses were performed from 0-degree gantry-tilt scans or from scans taken at other angles; new axial reconstructions with 0-degree tilt were generated. GE HealthCare AW Server software and Päijät-Häme Central Hospital radiologist workstations were used for the quantitative analysis according to the manufacturer’s instructions.

For statistical analysis to assess the effect of CT slice thickness on repeatability, we included patients with the same slice thickness in preoperative and postoperative CT and divided them into 2 groups: 1 mm or less (1 mm group) or more than 1 mm (2 mm+ group). Only the intact orbits were analysed. For the analysis, the difference between postoperative and preoperative volume was calculated. Because the distribution did not follow normality, the Shapiro-Wilk test was used; the medians of the calculated volume differences in each group were compared using the Mann-Whitney *U* test.

The agreement between observations from different methods was assessed using interclass correlation coefficients (ICC) for interrater reliability,^20^ and repeatability of each method was tested with intraclass correlation coefficients for intrarater reliability.^21^ The intact orbits and fractured orbits were analysed separately. For intraclass correlation, we used only intact orbits. The significance was set to *P*=0.05.

## RESULTS

Of 408 facial fracture patients, 190 patients suffered blow-out fractures, and 58 fulfilled the inclusion criteria. A further 24 were omitted due to insufficient imaging data for volumetric measurement, leaving 136 orbits from 34 patients for final analysis. The analysis of both preoperative and postoperative images allows the unaffected orbit to serve as its own control for the measurement comparison.

Demographic data for the patients are shown in Supplemental Table 1, Supplemental Digital Content 1, http://links.lww.com/SCS/J296. Thirteen patients were female, and 21 were male, and the average age was 45.0 years. Sixteen patients had fractures on the right side and 18 on the left side. Seven patients had pre- or postoperative CTs taken at angles other than 0 degrees, and thus had to be straightened for volumetric measurement.

For the slice-thickness analysis of automated segmentation, there were 14 patients in the 1 mm or less slice-thickness group and 12 in the 2 mm or more group. The difference in the median volumes was statistically significant (*P*=0.0288), showing that thinner CT slices are more reliable when using the automated segmentation tool. (Supplemental Table 2, Supplemental Digital Content 2, http://links.lww.com/SCS/J297).

Supplemental Table 3, Supplemental Digital Content 3, http://links.lww.com/SCS/J298 shows that repeatability in preoperative and postoperative intact orbits was highly reliable both in the manual and automated segmentation groups, ICC 0.83 and 0.92, respectively.

The volumetric correlation between radiologist-manual segmentation and automated segmentation was rather poor, whether in intact or fractured orbits, with interrater correlation ranging from 0.41 to 0.67. (Supplemental Table 4, Supplemental Digital Content 4, http://links.lww.com/SCS/J299).

## DISCUSSION

The aim of this study was to assess the repeatability and reliability of the automated segmentation tool. In the automated method, repeatability was significantly better when the slice thickness was 1 mm or less, or more than 2 mm. Thicker slices were the cause of the automated segmentation outliers. When measuring the volume of intact and fractured orbital vaults, our results show that both manual and automated methods are repeatable when assessing intact orbits in preoperative and postoperative CTs. The correlation between these different segmentation methods was rather poor both in intact and trauma orbits pre- and postoperatively.

The effect of slice thickness is not assessed in previous studies, but the automated segmentation process is reported earlier. A similar process in a previous study of 15 patients parallels our findings in repeatability of different measurements, although postoperative results had a wider range.^19^

Various methods to measure the volume of the orbit have been proposed. Direct method of filling (cadaveric) orbital vault with cast or beads or liquid was previously considered the gold standard.^22^ Filling of the cadaveric orbit may be applicable for scientific purposes, but naturally not for clinical, everyday diagnostics. Variation of that method, 3D printing and manual volume measuring from printed models have been proposed as well.^23^ In-house printing is fast enough, and 3D models may be repurposed for preoperative moulding of reconstruction mesh^16^ to reconstruct blow-out fracture within a reasonable time window. Anyways, this seems to add extra steps and time, and in the era of virtual surgical planning and PSI, the use of actual models seems unnecessary.^13,24^

Computed tomography (CT) is good for volumetric assessment and has been considered the gold standard for decades.^25^ Manual segmentation slice-by-slice is reliable but time-consuming and is dependent on the experience and personal assessment of the radiologists, and thus may not be reproducible if the same methods and criteria of measurements are not applied.^17^ Because of the conical structure of the orbital vault, defining the anterior boundary bears an important role in volume measurement. The orbital rim is described as a hyperbolic paraboloid structure, and even small differences may cause big errors.^22^ For this reason, a simultaneous zygomatic fracture makes the evaluation even more difficult. However, our study shows very good intrarater repeatability of manual segmentation, but the radiologist's experience is an important factor. Automated segmentation bypasses the difficulties in defining the anterior rims of the orbital vault,^19^ which may be a source of marked differences in manual segmentation.

Using automated segmentation is easy and straightforward; even a surgeon could learn it. Still, the volumetric results in different methods were not similar in our study. The explanatory factor may lie in the anterior opening: the automated segmentation model follows the anterior rim like a liquid with high surface tension, whereas the manual segmentation approximates boundaries as completely flat.

Increase in the orbital volume is 1 factor, but by no means the only factor in decision-making, primarily, whether to operate or not, and secondarily, if the operation is successfully accomplished.^4,26^ Immediate surgical intervention is usually needed in case of oculocardiac reflex, entrapment of the extraocular muscles, or marked retrobulbar hematoma. Severe enophthalmos or hypoglobus are unlikely to resolve over time, but oedema can mask diplopia and enophthalmos in the primary clinical assessment, and CT is a valuable tool in decision-making. Enophthalmos can result in compromised aesthetics, as well as gaze-evoked diplopia, eyelid retraction, lagophthalmos, and exposure keratitis.^27^ The volume change of the bony orbital vault is a risk factor in enophthalmos, but not the only reason for it. Enophthalmos correlates with volume change,^6,7^ but posterior volume seems to affect the most.^28^ Also, some studies suggest that fracture site is a more important factor as medial wall fractures need surgical involvement rather seldom even if volume is enlarged.^29^ Volume increase of 1 to 2 mm 3 has been proposed as a cutoff value for enophthalmos, and enophthalmos of more than 2 mm has been suggested as clinically significant.^30^ In our study, median volume differences in different methods were higher than that, showing the clinical relevance of our findings.

Orbits in normal population are rather symmetric,^8–11^ and in blow-out fracture surgery, postoperative results should reflect the contralateral side, but some overcorrection may be needed due to soft tissue atrophy.^12–14^

The retrospective nature of this study is the major limitation. Imaging protocols were not standardized, and pre- and postoperative CT scans may have been obtained on different scanners. In trauma CTs, patients may not be properly aligned; in this study, 7 CTs were straightened after measurement. This may further weaken this study. Also, the terms “semi-automated” and “automated” segmentation are not solid in the literature. In our study, we use the term automated because the process, although supervised by a radiologist, cannot be corrected or defined, even when the volume overestimation is obvious. Technical information on different systems is not clearly described in the literature, so comparisons are difficult.

A database of orbital volumes assessed with different methods should be collected and would be useful, as different methods give different results, if no agreement on volumetric assessment is reached. However, automating time-consuming parts of diagnostic processes is an emerging field in medicine and should be assessed with an open mind in the current trend toward more efficiently meeting medical needs.

To conclude, thinner CT slices provide more accuracy in automated volume assessment. For this reason, a slice thickness of <1 mm is recommended for imaging, especially if patient-specific reconstruction is considered. Automated and manual segmentation methods are repeatable for assessing the volume of the orbit, whether intact or fractured, but are not interchangeable and should not be interpreted as such.

## Supplementary Material
