# Supplementary material for: Volumetric Assessment of Blow-Out Fractures With Automated Segmentation Benefits Thinner Computed Tomography Slice Thickness: A Retrospective Case-Control Study
Source: J Craniofac Surg. 2026 Apr 13;37(7):1976–9. doi: 10.1097/SCS.0000000000012681 (PMC13290033; doi:10.1097/SCS.0000000000012681)
Supplement: Supplementary file 1 [file scs-37-1976-s001.docx]

*Supplemental table 1.* Demographics of the patients.

*Age (Years, mean) Sex Fracture site*

*45.0 13 (38.24%) female; 16 (47.1%) right;*

*21 (61.76%) male 18 (52.9%) left*
