# Supplementary material for: Volumetric Assessment of Blow-Out Fractures With Automated Segmentation Benefits Thinner Computed Tomography Slice Thickness: A Retrospective Case-Control Study
Source: J Craniofac Surg. 2026 Apr 13;37(7):1976–9. doi: 10.1097/SCS.0000000000012681 (PMC13290033; doi:10.1097/SCS.0000000000012681)
Supplement: Supplementary file 2 [file scs-37-1976-s002.docx]

*Supplemental table 2. The effect of slice thickness in automated segmentation measurements.*

*Slice thickness Number of patients Median CI*

*1mm or less 14 -0.1465 -0,513 – 0.0172*

*2mm or more 12 0.7285 0.003 – 1.056*

*p=0.0288**

*CI, lower and upper limits of 95% confidence interval. *p<0.05, significant.*
