# Supplementary material for: Volumetric Assessment of Blow-Out Fractures With Automated Segmentation Benefits Thinner Computed Tomography Slice Thickness: A Retrospective Case-Control Study
Source: J Craniofac Surg. 2026 Apr 13;37(7):1976–9. doi: 10.1097/SCS.0000000000012681 (PMC13290033; doi:10.1097/SCS.0000000000012681)
Supplement: Supplementary file 3 [file scs-37-1976-s003.docx]

*Supplemental table 3. Intrarater correlation in the pre- and postoperative volume of intact orbits.*

*Segmentation method ICC CI*

*Manual 0.83 0.69 – 0.91*

*Automated 0.92 0.85-0.96*

*ICC, intrarater correlation coefficient. CI, lower and upper limits of 95% confidence interval.*
