# Supplementary material for: Volumetric Assessment of Blow-Out Fractures With Automated Segmentation Benefits Thinner Computed Tomography Slice Thickness: A Retrospective Case-Control Study
Source: J Craniofac Surg. 2026 Apr 13;37(7):1976–9. doi: 10.1097/SCS.0000000000012681 (PMC13290033; doi:10.1097/SCS.0000000000012681)
Supplement: Supplementary file 4 [file scs-37-1976-s004.docx]

*Supplemental table 4. Inter-rater correlation in manual versus automated segmentation and median volumes (cm3).*

*Group AC1 CI Manual Automated*

*Preoperative intact 0.57 0.32 – 0.75 24.68 28.697*

*Postoperative intact 0.5 0.20 – 0.71 24.26 28.11*

*Preoperative trauma 0.41 0.11 – 0.64 27.3 30.68*

*Postoperative trauma 0.67 0.44 – 0.82 23.61 26.822*

*AC1, inter-rater correlation. CI, lower and upper limits of 95% confidence interval.*
